# Inter-Limb Asymmetry: Lessons Learnt from 10 Years of Data Collection in Performance Sport

**DOI:** 10.5114/jhk/224732

**Published:** 2026-07-19

**Authors:** Chris Bishop, Jose Afonso, Anthony Turner

**Affiliations:** 1 London Sport Institute, Faculty of Science and Technology, Middlesex University, London, UK.; 2 Centre of Research, Education, Innovation, and Intervention in Sport, Faculty of Sport, University of Porto, Porto, Portugal.

**Keywords:** side-to-side differences, performance sport, injury rehabilitation, athletic performance

## Abstract

For years, inter-limb asymmetry data have been reported in performance science and injury rehabilitation literature, together with routine monitoring in professional practice. Intuitively, many practitioners are almost ‘hard-wired’ to think that if a sizable inter-limb asymmetry presents itself, then something must be done to correct it. However, before any attempt is considered regarding trying to alter the magnitude of asymmetry, practitioners and researchers should first better comprehend the associated challenges and pitfalls of inter-limb asymmetry data. Once these are understood, a series of robust and pragmatic principles are proposed to enhance the utility of asymmetry data, which in turn, should help ensure that appropriate due-diligence is undertaken, serving to guide subsequent decision-making when considering this metric.

## Introduction

**Figure 1:**
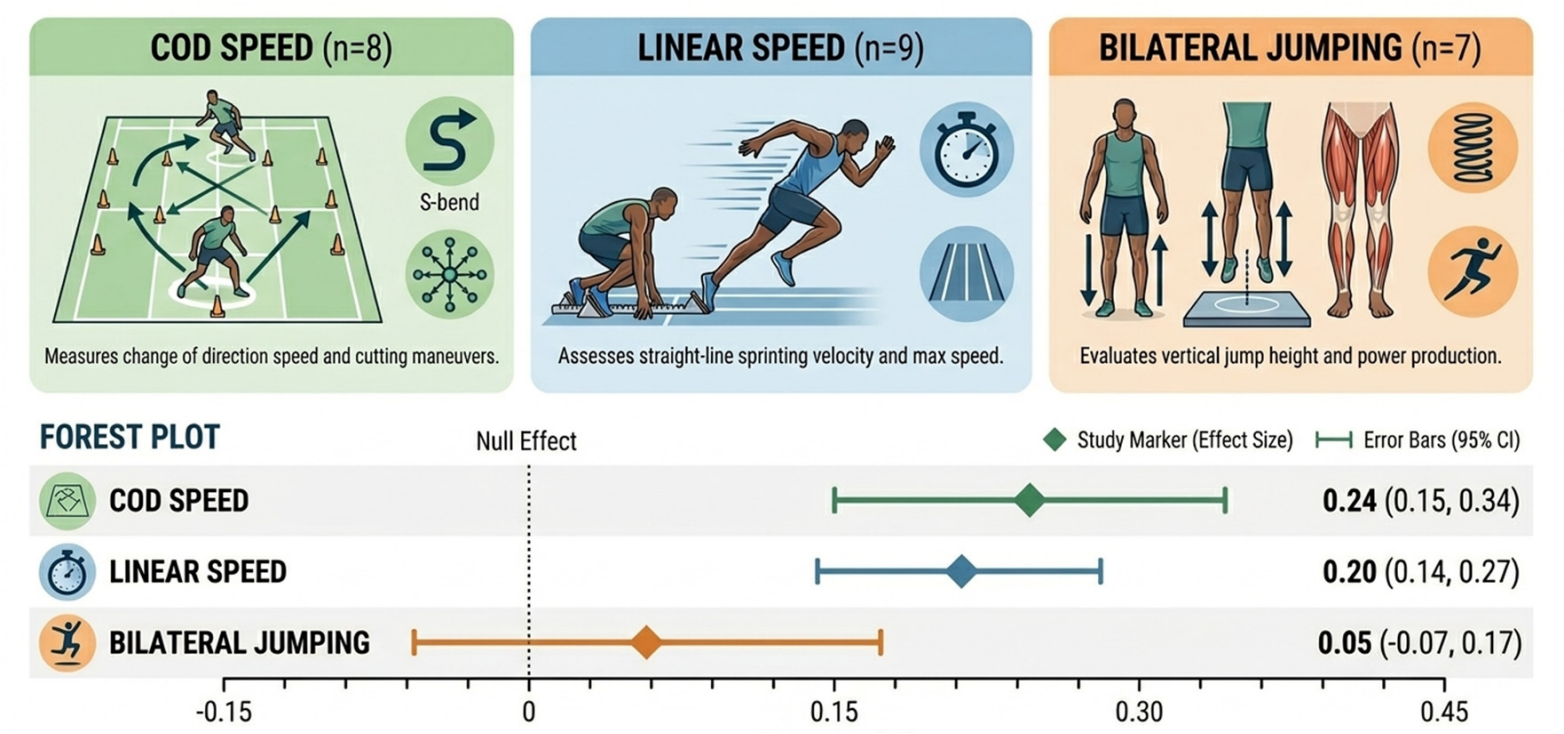
Summary infographic showing the effects (Fisher’s Z_r_) of inter-limb asymmetry on measures of change of direction speed, linear speed, and jumping. Data taken from Fox et al. (2023) and illustration created by ChatGPT.

For decades, inter-limb asymmetry data have been reported in performance science and injury rehabilitation literature, together with routine monitoring in professional practice. Intuitively, many practitioners are almost ‘hard-wired’ to think that if a sizable inter-limb asymmetry presents itself, that something must be done to correct it. Previous conceptual articles have outlined that collecting and utilizing physical capacity data should be grounded on a theoretical link to athletic performance or injury risk in said physical capacity (Bishop et al., 2022a; Turner 2024). When this principle is applied to asymmetry and performance, a plethora of articles have examined associations with independent measures of athletic or physical attributes (Bishop et al., 2018a, 2021, 2022b; Fox et al., 2023). However, the collective body of literature indicates that the effects of inter-limb asymmetry on measures of jumping, sprinting, and changing direction are small at best (Fox et al., 2023; Loturco et al., 2019) (Figure 1).

When focused on injury rehabilitation, numerous articles report limb symmetry (or asymmetry) data at different stages of an athlete’s rehabilitation journey (King et al., 2019; Kotsifaki et al., 2023; Kyritsis et al., 2016). However, recent narrative articles have suggested that the link between asymmetry and injury is rarely examined in a robust manner (Afonso et al., 2025, 2026). For example, problems include (but are not limited to) i) an over-reliance on cross-sectional data that shows association, not causation; ii) excessive focus on surrogate outcomes that may not actually be risk factors; iii) generalization of findings based on small samples; iv) few assessment points over time (i.e., unclear whether changes are real or reflect natural fluctuations); v) neglecting assessment errors that often are sufficient to explain away differences in assessments; and vi) framing the findings in a way that is not grounded on the data itself (i.e., spin). Even if these problems could be ignored, a systematic review examining the link between asymmetry and injury risk showed that approximately one third of included articles determined that some risk was evident, while another third showed no risk, and the remaining third were inconclusive (Helme et al., 2021). Thus, although restoring physical capacity remains an obvious priority during an athlete’s rehabilitation from injury, which in turn, is likely to reduce an imbalance over time, the consistent and repeatable link between a capacity imbalance and either performance or injury risk remains highly questionable.

Despite the challenges that the scientific literature has thrown up, many researchers and practitioners continue to chase asymmetry as an important metric to investigate, as shown by the volume of studies undertaken on this topic in the last 10–12 years (Becerra-Patino et al., 2025). Thus, given the inherent interest that appears to exist in this metric, the purpose of this commentary is twofold: i) to make researchers and practitioners aware of some of the historical issues with how asymmetry has been utilized, and ii) to offer a series of principles for practitioners and researchers to follow, helping to ensure that due-diligence is applied before utilizing inter-limb asymmetry data to inform subsequent decision-making.

## Historical Issues with Inter-Limb Asymmetry Data

Before outlining important information to enhance the utility of asymmetry data, we must first understand what some of the challenges are with the data. Put simply, we should not focus on the solutions without first understanding the problems and challenges that both practitioners and researchers face. The points below represent a brief synopsis of some historical issues that the authors have noted either from research or when using inter-limb asymmetry data in practice:

- **Calculation methods**. Numerous articles have illustrated that since the early 1980’s, approximately 10 different mathematical equations have been used to quantify inter-limb asymmetry (Bishop et al., 2016, 2018b; Parkinson et al., 2021). Up until 2016, almost all empirical research had ignored whether an equation was appropriate and, oftentimes, simply cited another study published beforehand as a loose justification for its use. Thus, there is some consideration that should be given for the mathematical formula which is selected to quantify existing side-to-side imbalances.
- **The problem with ratios**. The relevance of this issue is highlighted by recent research indicating that ratio data almost always exhibit greater ‘noise’ or measurement error (Bishop, 2025). This is because ratios are made up from two (and sometimes three) component parts, each of which exhibits its own level of error. Consequently, when an equation creates one single value (often a percentage) from multiple component parts, it essentially magnifies the error (Bishop et al., 2023). While this situation is far from ideal, the real problem becomes particularly relevant when practitioners are trying to track change over time and distinguish between differences inside or outside the error of the test measure.
- **Studies have often focused on outcome-measures only**. Until relatively recently, and particularly in the case of injury-based studies reporting asymmetry in test batteries, research has primarily reported inter-limb differences for outcome measures only. Hop testing has been especially prevalent in injury-based research, with side-to-side differences in hop distance commonly reported (Barber et al., 1990; Noyes et al., 1991; Kyritsis et al., 2016; Rohman et al., 2015). However, numerous papers have shown that outcome measures alone (especially in jumping) often miss the most relevant part of the test from both a performance (Bishop et al., 2022a; Gathercole et al., 2015) and injury rehabilitation (Kotsifaki et al., 2020, 2022) perspective.

**Asymmetry thresholds**. A number of studies have suggested that both athlete and general populations are at risk of increased injury if their inter-limb asymmetry is >15% (Barber et al., 1990; Noyes et al., 1991), and more recently, >10% (Kyritsis et al., 2016; Rohman et al., 2015). However, with an abundance of literature outlining that asymmetry is a highly task-specific measure, this feels like an inherently flawed concept. Put simply, if we know that a test is unlikely to produce the same asymmetry score between sessions or that multiple metrics from the same test will result in differing asymmetry values, how can one universal threshold be a predictor for anything (performance or injury) when its repeatability is almost non-existent.

Now that an overview has been provided regarding some of the challenges and flaws associated with asymmetry data, a set of five principles are proposed to help overcome these challenges, which, if adhered to, will help to enhance the utility of asymmetry data in practice.

## The 5 Principles to Follow for Utilizing Inter-Limb Asymmetry Data

**Figure 2:**
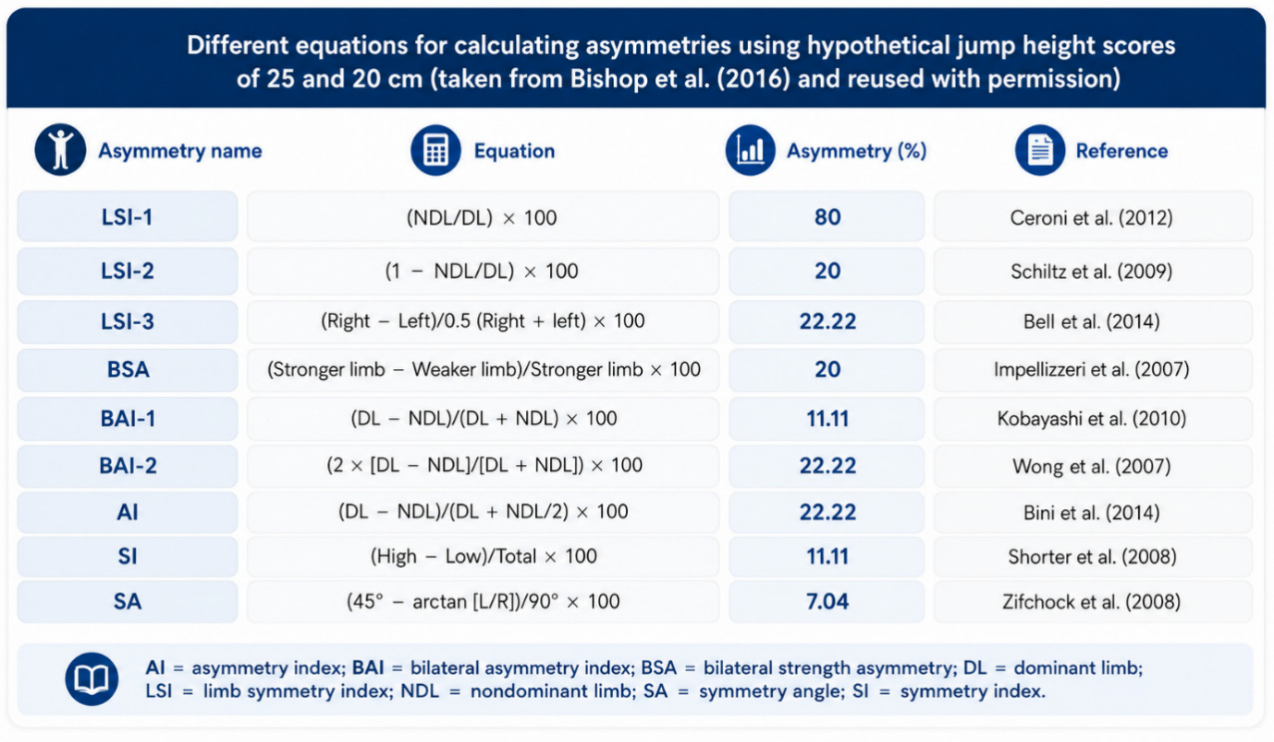
Hypothetical jump height data from a unilateral countermovement jump and the mathematical equations that have been used to calculate subsequent asymmetry outcomes. Data taken and adapted from Bishop et al. (2016) and created by ChatGPT.

**Figure 3:**
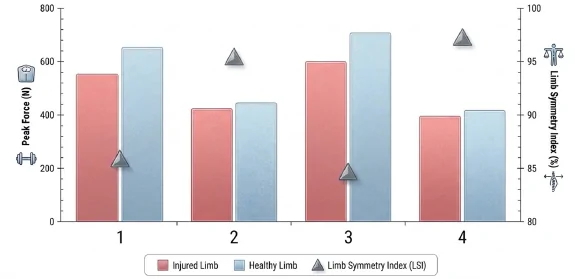
Hypothetical peak force and limb symmetry index data for four injured athletes. Image created by ChatGPT.

**Figure 4:**
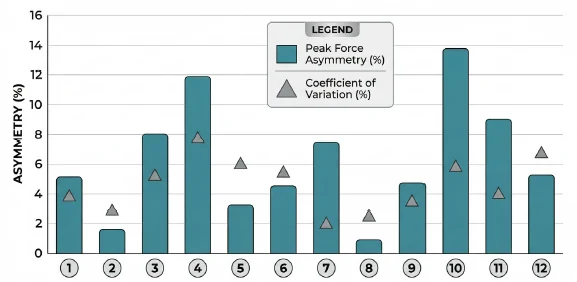
Hypothetical peak force asymmetry data and corresponding coefficient of variation values for peak force. Image created by ChatGPT.

**Figure 5:**
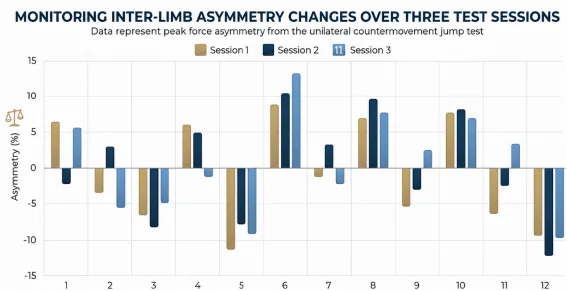
Hypothetical peak force asymmetry data from a unilateral countermovement jump over three consecutive test sessions. Image created by ChatGPT.

**Figure 6:**
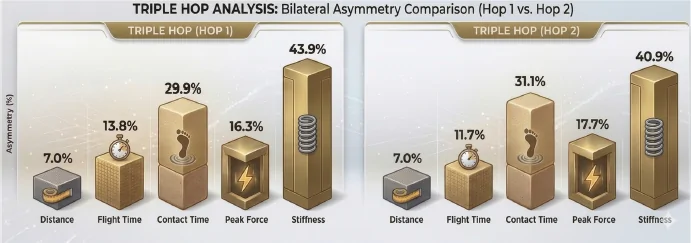
Hypothetical triple hop data for an injured athlete who previously suffered an anterior cruciate ligament rupture. Image created by ChatGPT.

1. **Get the math right**. An opinion piece from Bishop et al. (2018b) outlined a broad range of mathematical equations that have been used to quantify inter-limb asymmetries as a relative percentage. Importantly, this article outlined two key points for consideration. First, fundamental mathematical principles tell us that quantifying a relative percentage difference is the same as computing a fraction—of which we (as school children) are only taught one method. Thus, with asymmetry also being a relative percentage difference, it is challenging to understand why fundamental math principles should not be adhered to. However, and in contradiction to this viewpoint, the second point was that quantifying asymmetry from bilateral and unilateral tasks may not be the same. Put simply, Figure 2 provides hypothetical jump height scores from a unilateral countermovement jump. If we accept that the calculation must follow the principles of computing fractions, then the correct answer is the ‘BSA equation’ in row four. It is important to note as well that readers may view the data in Figure 2 and think that ‘LSI-1’ or ‘LSI-2’ can also be used, given they produce the same 20% outcome. However, these equations do not stand the test of time because there is no guarantee that the dominant limb will always score higher. Thus, practitioners and researchers can always trust that the BSA formula will adhere to those aforementioned important mathematical principles. As a final point of consideration, the only time practitioners may wish to consider an alternative formula is during bilateral tests. For example, if limb differences in peak force were being quantified from a bilateral countermovement jump, it makes more sense to quantify the imbalance relative to the total force output, noting that both legs interact together to produce a sum force value. In this instance, both the ‘BAI-1’ and ‘SI’ equations can be the formulas to select.
2. **Use an appropriate benchmark**. The data in Figure 3 illustrate hypothetical peak force data on both limbs in four injured athletes, along with their subsequent limb symmetry index scores. As mentioned in the ‘historical issues’ section, numerous studies have suggested binary thresholds which are indicative of increased injury risk. If the blanket 10% threshold was applied to the data shown in Figure 3 (remembering that there is no reasonable scientific basis for this proposed value), athletes 1 and 3 would be at a greater risk of injury than athletes 2 and 4. However, athletes 2 and 4 have the lower peak force scores. Thus, the conundrum then becomes whether superior capacity or symmetry is more desirable. Given the evidence for superior strength being one of the most important physical capacities for mitigating the risk of injury (Lauersen et al., 2014, 2018), it seems prudent to suggest that in this scenario, athletes 2 and 4 are likely to be the ones at greater risk. In further support of this, more recent evidence suggests that pre-injury capacity levels are more sensitive at detecting whether athletes are ready to return to training over limb symmetry alone (Wellsandt et al., 2017). Thus, practitioners are encouraged to carefully consider the most appropriate benchmark when determining whether an athlete is ready to return to training and competition. Furthermore, if practitioners decide to monitor asymmetry data, they must do so alongside the component parts.
3. **Importance of signal to noise**. Another critical point to enhance the utility of asymmetry data relates to its interpretation relative to the raw data’s measurement error. Exell et al., (2011) examined gait asymmetries and determined that a side-to-side difference was only considered ‘real’ if it was greater than the intra-limb variability or measurement error. When this principle is viewed in respect to the hypothetical data in Figure 4, those athletes with a peak force asymmetry (blue bar) greater than the coefficient of variation (CV) for peak force (grey triangle) are exhibiting a true asymmetry. In contrast, when the CV value sits higher than the blue bar, the magnitude of asymmetry is less than the error of the test measure and thus, not real. This becomes a critical part of filtering whether subsequent asymmetry scores even warrant any further consideration. Put simply, if an asymmetry value is greater than the CV, but the CV is considered too high (i.e., >10%) (Banyard et al., 2017), then the subsequent imbalance score can likely be ignored due to the measurement error of the metric in question being too high (and therefore, unreliable). It is important to note that an asymmetry being ‘real’ does not automatically mean practitioners need to try and remedy it. Rather, understanding how asymmetry stacks up against measurement error serves as an initial ‘spot check’ for practitioners to either disregard data or keep it on their radar for further discussions and monitoring.
4. **Magnitude vs. direction**. As previously mentioned, asymmetry is a ratio, and for too long, many empirical studies have treated it as a single value, almost in the same way as the raw scores themselves. The relevance here is that for asymmetry to be present, one limb must produce a superior score compared to the other. Figure 5 illustrates hypothetical data on peak force asymmetry from a unilateral countermovement jump in 12 athletes over three testing sessions. This time, however, the data are presented with directionality included, whereby positive values are indicative of the right limb producing more force and negative values are indicative of the left limb producing more force. When data are viewed this way, it illustrates a couple of important considerations—both of which will be articulated by selecting specific example athletes from the graph. Firstly, athlete 1 starts with a right-limb dominant asymmetry of 6.5%, but at the next time point they have an asymmetry of −2.0% and are now left-limb dominant. If practitioners only monitored the absolute percentage value (i.e., always as a positive value with no directionality considered), they might mistakenly believe that an asymmetry that shifts from 6.5% to 2.0% is a positive change. However, this change is not so much a 4.5% reduction in asymmetry as it is an 8.5% ‘shift’, and if practitioners do not monitor directionality, this will be missed. Second, if we consider athletes 5, 6, or 12, we can see that they depict consistent directionality at each time point. Whilst practitioners should be mindful of any pre-determined thresholds and their inability to be predictive or causative in nature, it seems fair to suggest that if an athlete has a consistently under-performing or weaker limb, this may warrant some further discussion or consideration. For example, practitioners may wish to consider whether this represents a functional imbalance as a consequence of the demands of the athlete’s sport, whether it is a residual deficit from a prior injury, or whether it cannot be fully explained. To some extent, and regardless of the reason, if practitioners give a weaker limb improvements in capacity, this is unlikely to result in detrimental side effects. As such, these scenarios in which athletes depict consistent directionality may represent legitimate ‘windows of opportunity’ for enhanced capacity development (Maloney, 2019).
5. **Include movement strategy metrics**. The final point for practitioners to consider is the inclusion of metrics beyond outcome measures in their assessment protocols. Work from Kotsifaki and colleagues (2020) critiqued hop distance as being insufficient for detecting deficits in knee function after anterior cruciate ligament (ACL) injury. In addition, the subsequent asymmetry value from hop distance has also been heavily scrutinized as masking potentially more relevant between-limb biomechanical deficits in knee function (Kotsifaki et al., 2022). To illustrate this point, Figure 6 exhibits hypothetical asymmetry data for a range of metrics during a triple hop for distance test in a single athlete who has previously suffered an ACL rupture. The chosen metrics are in line with a study by Davey et al. (2022) and data are provided between hops 1 and 2 (left half of the image) and hops 2 and 3 (right half of the image). Important to note, because distance represents the outcome measure at the end of the assessment, these values are the same in both graphs. Equally, if we were to consider this score relative to previously suggested thresholds of 10–15%, then this athlete “might” be deemed fit to return to training. However, when viewing some of the other metrics, it is clear to see that some large between-limb deficits still exist—especially for contact time and stiffness. In addition, and perhaps the most relevant point of all, if this athlete experienced a previous ACL rupture, then metrics such as contact time and stiffness are likely to be more relevant proxies reflecting the athlete’s ability to attenuate force on landing—a concept known to be a risk factor for this type of injury (Della Villa et al., 2020; Montgomery et al., 2018). Thus, outcome measures alone give athletes a chance to falsely prove they are ready to return to training, whereas relevant movement strategy metrics are likely to be more informative, perhaps in both performance and rehabilitation settings.

**Figure 7:**
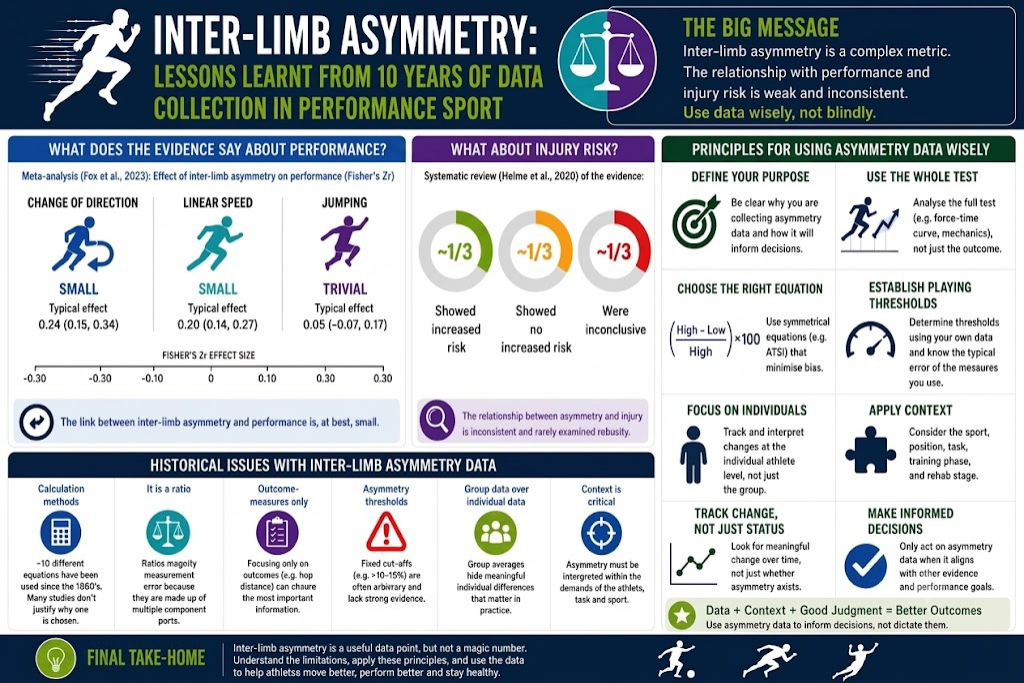
Summary infographic outlining associations between asymmetry, performance, and injury risk, key historical challenges and subsequent principles to follow to enhance data utility. Image created by ChatGPT.

## Conclusions

In summary, inter-limb asymmetry presents routine challenges for practitioners given it is a noisy ratio metric, which, in turn, is a contributory factor to weak and complex relationships with both performance and injury risk. For some, this may be reason enough to disregard its use entirely. However, some interesting discussions can be garnered from asymmetry data if practitioners have a pertinent understanding of the associated mathematics, select an appropriate benchmark from which to help guide decision-making, and consider the associated measurement error, directionality, and relevant metrics from the assessment protocol. In light of the complex inter-play between historical issues and proposed principles to enhance asymmetry data utility, a summary infographic has been provided to aid with knowledge dissemination for the reader (Figure 7).
